# Microorganisms and Biostimulants Impact on the Antioxidant Activity of Buckwheat (*Fagopyrum esculentum* Moench) Sprouts

**DOI:** 10.3390/antiox9070584

**Published:** 2020-07-04

**Authors:** Robert Witkowicz, Wioletta Biel, Edyta Skrzypek, Joanna Chłopicka, Katarzyna Gleń-Karolczyk, Mateusz Krupa, Ewelina Prochownik, Agnieszka Galanty

**Affiliations:** 1 Department of Agroecology and Crop Production, University of Agriculture in Krakow, Mickiewicza 21, 31120 Krakow, Poland or robert.witkowicz@urk.edu.pl (R.W.); mateuszkrupa1990@gmail.com (M.K.); 2 Department of Monogastric Animal Sciences, Division of Animal Nutrition and Food, West Pomeranian University of Technology in Szczecin, 29 Klemensa Janickiego Street, 71270 Szczecin, Poland; 3 The Franciszek Górski Institute of Plant Physiology, Polish Academy of Sciences, Niezapominajek 21, 30239 Krakow, Poland; e.skrzypek@ifr-pan.edu.pl; 4 Department of Food Chemistry and Nutrition, Medical College, Jagiellonian University, Medyczna 9, 30688 Krakow, Poland; joanna.chlopicka@uj.edu.pl (J.C.); ewelina.gajdzik@uj.edu.pl (E.P.); 5 Department of Microbiology and Biomonitoring, University of Agriculture in Krakow, Mickiewicza 21, 31120 Krakow, Poland; rrglen@cyf-kr.edu.pl; 6 Department of Pharmacognosy, Medical College, Jagiellonian University, Medyczna 9, 30688 Krakow, Poland; agnieszka.galanty@uj.edu.pl

**Keywords:** antioxidant activity, *Bacillus subtilis*, buckwheat sprouts, *Ecklonia maxima*, natural nitrophenols, *Pythium oligandrum*

## Abstract

The study analyzes the influence of plant growth promoters and biological control agents on the chemical composition and antioxidant activity (AA) in the sprouts of buckwheat. The AA of cv. Kora sprouts was higher than cv. Panda, with 110.0 µM Fe^2+^/g (FRAP—Ferric Reducing Antioxidant Power), 52.94 µM TRX (Trolox)/g (DPPH—1,1-diphenyl-2-picrylhydrazyl), 182.7 µM AAE (Ascorbic Acid Equivalent)/g (Photochemiluminescence—PCL—ACW—Water-Soluble Antioxidant Capacity) and 1.250 µM TRX/g (PCL—ACL—Lipid-Soluble Antioxidant Capacity). The highest AA was found in the sprouts grown from seeds soaked in *Ecklonia maxima* extract and *Pythium oligandrum* (121.31 µM Fe^2+^/g (FRAP), 56.33 µM TRX/g (DPPH), 195.6 µM AAE/g (PCL—ACW) and 1.568 µM TRX/g (PCL—ACL). These values show that the antioxidant potential of buckwheat sprouts is essentially due to the predominant hydrophilic fraction of antioxidants. The AA of the sprouts was strongly correlated with total polyphenol content.

## 1. Introduction

Enlargement of the food industry raw material base opens up new possibilities in diversifying the human diet, minimizing nutrient deficiencies, and protecting against chronic diseases [1]. According to a WHO report [2], 71% of deaths worldwide were caused by noncommunicable diseases (cardiovascular disease, cancer, chronic respiratory disease, diabetes). The observed increase in these diseases and acceleration of human body’s aging processes are associated with the activity of free radicals, causing damage to cellular organelles. A natural solution to reduce the free radicals’ harmful effects in the organism is an antioxidant-rich diet comprising of fruits, vegetables, cereals and pseudocereals, grains and their sprouts. The popularity of sprouts of different species is a result of their visual and sensory attractiveness and appreciable content of bioactive substances [3,4].

Jing et al. [5] indicated species of the genus *Fagopyrum* as valuable edible and medicinal plants, with over 100 compounds belonging to six classes (flavonoids, phenolic acids, fagopyritols, triterpenoids, steroids, and fatty acids) described so far. Moreover, *Fagopyrum* sprouts have been extensively studied recently, with *Fagopyrum esculentum* sprouts being the most popular and containing valuable flavonoids [6,7,8]. The metabolism of the sprouts can be additionally modified to obtain more intensive growth or increased concentration of bioactive components [9,10,11,12]. This was achieved by inoculating the seeds with *Herbaspirillum* sp. [13] or *Fusarium oxysporum* Fat9 [14] mycelium extract, while treatment of rice seeds with *Bacillus subtilis* resulted in an increased length of the root and seedling stem [15]. In our previous work we have proven that soaking *Fagopyrum esculentum* seeds in a solution of *Ecklonia maxima* extract promoted the accumulation of sprouts dry matter (DM), while the treatment with *Bacillus subtilis* resulted in a decrease in the dry matter level in the sprouts, enabling the production of sprouts with the lowest crude fiber content. Moreover, the use of a *Pythium oligandrum* oospores solution for seeds soaking reduced the ash level in the sprouts, while the addition of nitrophenols increased the level of both ash and proteins [16].

According to Frederiks and Wesseler [17], 48 microbial biological control agents (BCAs) are now registered in Europe, including fungi, oomycetes, and bacteria (e.g., *Bacillus subtilis* str. QST 713, *Bacillus thuringiensis* subsp. *israeliensis* (serotype H-2014) strain AM65-52, *Pythium oligandrum* M1, *Pseudomonas chlororaphis* str. MA342, *Streptomyces* K61, *Candida oleophila* str. O, *Saccharomyces cerevisiae* str. LAS02). Natural biostimulants have similar impact on plants and are known as plant growth promoters (PGPs) [18,19,20,21,22], and elicitors [11]. The group is very heterogeneous and consists of humic substances, organic material, beneficial chemical elements, inorganic salts, seaweed extracts, chitin and chitosan derivates, free amino acids, and N-containing substances. 

The study aimed to determine the optimal combination of BCAs and/or PGPs treatment of *Fagopyrum esculentum* seeds for obtaining the sprouts with better nutritional value and medicinal potential. Thus, in the study we have analyzed if the selected BCAs (*Pythium oligandrum* oospores and *Bacillus subtilis*) and/or PGPs (*Ecklonia maxima* extract and mixture of three nitrophenolic compounds) can influence the antioxidant activity of buckwheat sprouts, and the content of rutoside, total polyphenols, and organic acids.

## 2. Materials and Methods

### 2.1. Plant Material, Experiment Set-Up, and Sample Preparation

Common buckwheat (*Fagopyrum esculentum* Moench) seeds were purchased from the Plant Breading of Agricultural Crops Experimental Station Palikije (Poland). The experiment was established at room temperature (ca. 21 °C) with the presence of the natural light. The buckwheat seeds were soaked in different treatment solutions for 30 min. The treatment solutions of different PGPs and BCAs were prepared according to the labels attached by manufacturers. Bioregulator Kelpak SL (Kelp Products International (Pty) Ltd., Simon’s Town, South Africa) is a commercial product (seaweed *Ecklonia maxima* extract) and contains polysaccharides laminarin, alginates and carrageenans, micro- and macronutrients, sterols, N-containing compounds like betaines and hormones. The 1:100 treatment solution of Kelpak SL was used. Biostimulant Asahi SL (Arysta LifeScience Ltd., Praha, Czech Republic) is a commercial product and contains a mixture of three nitrophenolic compounds (sodium p-nitrophenolate, sodium o-nirtophenolate, sodium 5-nitroguaiacolate). The 1:500 treatment solution of Asahi SL was used. Polyversum WP (Biopreparaty Ltd., Uherce, Czech Republic) is a commercial product and contains 10^6^ fungi *Pythium oligandrum* oospores in 1 g. The treatment solution contains 2 g of Polyversum WP in 500 mL of water. Serenade ASO (Bayer AG, Leverkusen, Germany) is a commercial product and contains *Bacillus subtilis* QST 713–13.96 g/L (1.34%) (minimum number of bacterial cells 1.042 × 10^12^ CFU/L). The 1:40 treatment solution of Serenade ASO was used. The treatment solutions combinations were: 1. Kelpak SL only, 2. Asahi SL only, 3. Polyversum WP only, 4. Serenade ASO only, 5. Kelpak SL plus Polyversum WP, 6. Asahi SL plus Polyversum WP, 7. Kelpak SL plus Serenade ASO, 8. Asahi SL plus Serenade ASO, 9. Asahi SL plus Kelpak SL and 10. Control (untreated, without biostimulants and BCAs). After the treatment, the seeds were washed in distilled water, placed in the sprouting plastic plate, and watered once a day. The harvest was done 14 days after seeding (DAS). The buckwheat sprouts were immediately dried in 40 °C. Dry buckwheat sprouts were ground into powder in the laboratory mill (type KNIFETEC 1095, Foss Tecator, Höganäs, Sweden).

To make the manuscript more reader-friendly, we will use the term “sprouts pretreated with…” instead of a more precise but longer term “the sprouts grown from the seeds treated with…” throughout the text.

### 2.2. Extracts Preparation

Dry pulverized samples (1 g) were extracted with 95% ethanol during 2 h, and the extracts were stored in refrigerator (−20 °C) and further used for ferric reducing antioxidant power (FRAP), 1,1-diphenyl-2-picrylhydrazyl (DPPH), TPC and PCL—ACW assays. Extracts for PCL—ACL assay were prepared from 0.2 g samples extracted with 3 mL hexane for 2 h.

### 2.3. Determination of Antioxidant Activity

#### 2.3.1. Ferric Reducing Antioxidant Power (FRAP) Assay

Ferric reducing antioxidant power (FRAP) was estimated using the procedure described by Benzie and Strain [23]. The aliquots of ethanolic extracts were mixed with 0.01 M 2,4,6-Tri(2-pyridyl)-s-triazine (Fe(III)/TPTZ) reagent in 0.3 M acetate buffer (pH 3.6) and kinetic measurements at 593 nm were performed during 1 h on a microplate reader (Synergy 2, Bio-Tek, Winooski, VT, USA). Results were calculated for 4 and 60 min incubation and expressed in µM Fe^2+^/g DM.

#### 2.3.2. 1,1-Diphenyl-2-Picrylhydrazyl (DPPH) Assay

Total antioxidant activity was determined by 1,1-diphenyl-2-picrylhydrazyl (DPPH) assay as described by Brand-Williams et al. [24]. The aliquots of ethanolic extracts were mixed with 0.1 M Tris-HCL buffer (pH 7.4) and 0.2 mM DPPH solution, incubated at room temperature in the dark. The absorbance was measured at 517 nm 60 min. after addition of DPPH solution on a microplate reader (Synergy 2, Bio-Tek, Winooski, VT, USA). The Trolox equivalent was used to express antioxidant activity in µM Trolox/g DM.

#### 2.3.3. Photochemiluminescence (PCL) Assay

Extracts for photochemiluminescence (PCL) were performed according to Popov and Lewin [25]. The measurements were performed with Photochem^®^ Antioxidant Analyzer (Analytik Jena AG, Leipzig, Germany) using commercial kits as reagents. In the PCL method, photochemically generated superoxide anion radicals are eliminated by reaction with the antioxidants present in sample and after a time they were exhausted the remaining radicals produces luminescence allowing determination of the antioxidant capacity of the sample in lipid-soluble (ACL) and water soluble (ACW) fractions. Results were expressed as µM of Trolox equivalent per g DM for ACL fraction and as ascorbic acid equivalent (AAE) per g DM for ACW fraction.

#### 2.3.4. Determination of Total Polyphenols (TP) Content

Total polyphenols content was measured according to Singleton and Rossi [26] with Folin-Ciocalteu reagent. The aliquots of ethanolic extracts were mixed with 500 μL of deionised water, 250 μL of 25% (*w*/*v*) Na_2_CO_3_, and 125 μL of Folin-Ciocalteau reagent. The absorbance was measured at 760 nm on a microplate reader (Synergy 2, Bio-Tek, Winooski, VT, USA). The results were expressed in mg gallic acid equivalents (GAE) per g DM.

#### 2.3.5. Determination of Organic Acids Content

Organic acids content were analyzed according to Dobrowolska-Iwanek et al. [27] using the high performance liquid chromatograph (HPLC) LC20AD (Shimadzu, Tokyo, Japan) with DAD detector and Hydro-RP 80A (4 µm) column (250 × 4.6 mm), scanning range 200–350 nm, and flow rate ca. 1 mL per min. Mobile phase consisted of 1.4 mL 85% (*v*/*v*) phosphoric acid, 1 mL diethylamine, 20 mL acetonitrile, and deionised water to volume 1 L. The ethanolic extract of dry pulverised sprouts (0.5 mL) was dried with air flow at room temperature and 0.050 mL of methanol was added to a residue. After ca. 1 h deionized water was added to total volume of 1.5 mL and was shaken for 2 h. The solutions were filtered before analysis using syringe filter 0.22 µm. The following organic acids were used as standards: oxalic, malic, acetic, citric, malic, succinic, and fumaric. Identification of the compounds was conducted by comparing their retention time and spectrum with the standards (Merck KGaA, Darmstadt, Germany). Quantification of them was performed by measuring the peak area regarding the standard curve prepared from five concentrations (0–100 mg/L). The results were expressed as mg per 100 g DM.

#### 2.3.6. Determination of Rutoside Content

Rutoside content analysis was performed as described by Paśko et al. [28], using a Dionex HPLC system with a PDA 100 UV–visible detector Hypersil Gold (C-18) column (5 μm, 250 × 4.6 mm, Thermo EC) mobile phase of 1% (*v*/*v*) formic acid in water (A) and acetonitrile (B) in a gradient mode 5–60% B over 60 min., at a flow rate of 1 mL per min. The ethanolic extracts were filtered before analysis using syringe filter 0.45 µm. Identification of the compound was conducted by comparing its retention time and UV spectrum with the standard rutoside (Merck KGaA, Darmstadt, Germany). Quantification of rutoside was performed by measuring the peak area regarding the standard curve prepared from five concentrations (1—0.0625 mg/mL). The results were expressed as mg per 100 g DM. All measurements were performed in triplicate.

### 2.4. Statistical Analyses

The two factorial analyses of variance (ANOVA) were done [29]. The significance of differences between means shown in the form of homogeneous groups which was assessed using a Newman-Keuls’ test at *p* = 0.05. Principal component analysis (PCA) was done and presented as biplot [29].

## 3. Results and Discussion

The influence of PGPs and/or BCAs was also confirmed by statistical analysis (Table 1, Table 2 and Table 3). The antioxidant activity (AA) of the sprouts from Panda and Kora buckwheat cultivars was significantly modified by the pretreatment of the seeds with different combinations of PGPs and/or BCAs (Table 1). The results of AA determination of the tested buckwheat cultivars (expressed in FRAP, DPPH, PCL) showed unambiguously that Kora sprouts revealed stronger anti-free radical properties and also a higher FRAP ratio (FRAP 60′/FRAP 4′). The increase in AA from the 4th to 60th minute, was statistically higher in Kora sprouts when compared to Panda cultivar. The obtained data also revealed that the essential AA of buckwheat sprouts of both cultivars results from the activity of hydrophilic antioxidants (ACW). AA of the buckwheat sprouts, measured by FRAP method 60 min after the beginning of the redox reaction, revealed the same order of experimental combinations (seed treatment) as in the 4th minute of the measurement. 

The weakest AA was observed for the sprouts pretreated with *Ecklonia maxima* extract (39.83 and 83.03 µM Fe^2+^/g, for FRAP 4′ and 60′, respectively) and these values were significantly lower than those determined for the untreated sprouts (58.48 and 112.91 µM Fe^2+^/g, for FRAP 4′ and 60′, respectively). The combination of *Ecklonia maxima* extract and *Pythium oligandrum* oospores caused a rapid increase in the iron (III) reduction capacity, above the level determined in the control (62.47 and 121.31 µM Fe^2+^/g, for FRAP 4′ and 60′, respectively). In a study by Zhong et al. [14], who used four polysaccharides from the mycelium of *Fusarium oxysporum* Fat9 at six different concentrations, only three combinations induced a decrease in AA of the buckwheat sprouts using the DPPH^+^ radical, and one combination with the ABTS^+^ radical. Metabolic analysis of seven-day-old buckwheat sprouts performed by Kim et al. [30] revealed significant influence of methyl jasmonate on buckwheat sprouts. This elicitor increased AA from about 120 to about 250 µM Fe^2+^/g and proved to be much more effective in comparison to our study, where only one combination increased AA in buckwheat sprouts. In a study by Świeca [31], soaking buckwheat seeds in shikimic acid and/or amino acids, followed by spraying 1 day-old buckwheat sprouts with *Salix myrsinifolia* bark extract, yielded raw material with varied AA expressed as free radical scavenging ability (from 3.41 to 14.28 mg of TRX/g DM).

**Figure 1:**
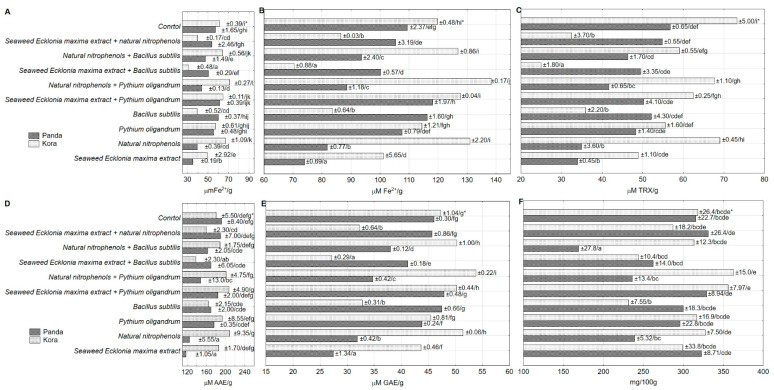
Effect of the treatment on antioxidant activity (Ferric Reducing Antioxidant Power (FRAP) 4′ (**A**), FRAP 60′ (**B**), 1,1-diphenyl-2-picrylhydrazyl (DPPH) (**C**), Water-Soluble Antioxidant Capacity ACW (**D**)), total polyphenols (**E**), and rutoside (**F**) content in the buckwheat sprouts cvs. Panda and Kora. * standard error of mean/homogeneous groups.

Treatment of the seeds with different combinations of PGPs and/or BCAs caused AA modification in the sprouts of the two examined buckwheat cultivars determined by the FRAP 4′ and FRAP 60′ (Figure 1A,B, respectively). The presence of nitrophenols mixture in the treatment solution caused an increase in AA in Kora sprouts but a decrease in Panda sprouts, in comparison to the untreated seeds. Treatment of the seeds with *Bacillus subtilis* alone or combined with *Ecklonia maxima* extract, resulted in a significant AA decrease in Kora sprouts, compared to the control. The influence of *Pythium oligandrum* oospores alone or combined with *Ecklonia maxima* extract resulted in a similar AA of both tested cultivars sprouts when compared to the control.

The FRAP 60′ to FRAP 4′ ratio ranged from 1.931 to 2.085, which means that the amount of reduced iron (III) doubled between the measurements. A slightly greater increase in reducing capacity was observed in Kora sprouts (Table 1) which may suggest higher contribution of fast reducers in this process, in comparison to Panda cultivar. The components present in the treating solutions influenced the value of FRAP ratio, observed as the smaller values of FRAP ratio at higher FRAP values (both in the 4′ and 60′). This was also confirmed by a negative correlation coefficient between AA determined at the 4th and 60th minute by FRAP method and the FRAP ratio (Table 3).

The AA of the buckwheat sprouts measured with DPPH^+^ radical was dependent on the composition of the solutions used to soak the seeds (Table 1). Due to the lipophilic nature of the DPPH^+^ radical, a slight difference in the order of experimental combinations (seed treatment) was observed in the AA measured by DPPH, in comparison to the FRAP assay. The highest AA determined by the DPPH^+^ radical was shown in the control sprouts. The presence of PGPs or BCAs in the solution resulted in a decrease in AA in the pretreated sprouts, with one exception of the combination of *Ecklonia maxima* extract and *Pythium oligandrum* oospores. The algae extract alone and in the presence of the nitrophenols reduced antioxidant activity.

The obtained results also indicated the varied influence of the tested PGPs and/or BCAs on AA of the two tested buckwheat cultivars. In three out of the ten tested combinations, AA was significantly lower in Kora than in Panda sprouts. This was observed for the treatment with *Bacillus subtilis* alone or in the combination with *Ecklonia maxima* extract, but also for the combination of the latter with nitrophenols mixture (Figure 1C).

Antioxidant activity determined by PCL-ACW method confirmed the observations from the total antioxidant potential analysis by FRAP. This indicates that buckwheat sprouts contained mainly antioxidants belonging to the hydrophilic fraction. The highest AA was noted for the untreated sprouts and the sprouts pretreated with two solutions containing *Pythium oligandrum* oospores (Table 1), while the lowest AA was observed for the sprouts pretreated with two solutions of *Ecklonia maxima* extract. The antioxidant activity of the lipophilic fraction (ACL) of buckwheat sprouts was generally low. The only increase in the AA dependent on lipophilic fraction was observed for the sprouts pretreated with the solution of *Ecklonia maxima* extract and *Pythium oligandrum* oospores (1.568 µM TRX/g). Colonna et al. [32] analyzed the AA of hydrophilic and lipophilic fractions of ten leaf vegetables. The results indicated that AA of the hydrophilic fraction ranged from 7.1 to 15.4 µM AAE/g fresh weight, while for the lipophilic fraction from 4.7 to 7.8 µM TRX/g fresh weight. In comparison to this, the AA of the hydrophilic fraction of the sprouts in our study was significantly higher, even taking into account the fact that it was given in dry mass of the raw material, ranging from 151.7 to 195.6 µM AAE/g, while the AA of the lipophilic fraction ranged only from 0.933 to 1.568 µM TRX/g DM.

The varied influence of the tested PGPs and/or BCAs on the AA of hydrophilic fraction was observed in the sprouts of the two studied cultivars (Figure 1D). The seeds treated with PGPs alone resulted in the sprouts of Panda cultivars with lower the AA of hydrophilic fraction than Kora sprouts, when compared to the untreated sprouts (both showed the same level AA of hydrophilic fraction). The presence of *Pythium oligandrum* oospores and PGPs increased the AA of hydrophilic compounds in Kora sprouts. On the other hand, the presence of *Bacillus subtilis* in the treating solution affected the tested cultivars differently, depending on the accompanying PGPs. In the presence of *Ecklonia maxima* extract, a higher AA of hydrophilic fraction was noted for Panda sprouts, while in the presence of nitrophenols mixture it was elevated in Kora sprouts.

The Total Polyphenols (TP) content showed an almost linear relationship with the AA determined by FRAP (Table 3). The value of the correlation coefficient was 0.96, regardless the time of AA determination (both at the 4th and 60th minute). The collinearity of these two parameters was disturbed only for the sprouts pretreated with *Bacillus subtilis* alone (Table 2). Despite the moderate TP content (40.16 mg GAE/g) the sprouts showed relatively high antioxidant activity measured by FRAP method at 4th and 60th minute (53.36 and 105.38 µM Fe^2+^/g, respectively). The relatively low correlation coefficient (0.6) between the content of rutoside in the sprouts and their AA measured by FRAP and DPPH methods (Table 3) may result from the presence of other polyphenolic compounds. Chłopicka et al. [33] confirmed the presence of 3-hydroxycinnamic, ferulic, caffeic, protocatechuic and syringic acids in the extracts of 7- and 10-day-old buckwheat sprouts. The levels of ferulic and syringic acids in 10-day-old buckwheat sprouts were only 0.01 mg/g DM, and the protocatechuic acid level was 0.05 mg/g DM. The caffeic acid levels did not change over time and for both dates it was 0.02 mg/g DM. The content of 3-hydroxycinnamic acid at day 10 of germination (0.94 mg/g DM) increased fourfold compared to day 7 of germination (0.23 mg/g DM). Compared to leafy vegetables (e.g., chicory, green and red lettuce, spinach) studied by Colonna et al. [32], the TP content in buckwheat sprouts in our study was significantly higher. In the study by Kreft et al. [34] the TP content in buckwheat sprouts of Darja cultivar ranged from 0.26–1.5% DM and in Tartary buckwheat from 2.0 to 3.3%. The TP content in Darja sprouts was then similar to the results obtained for Panda and Kora sprouts in our study. Yang et al. [35], observed an increase in the TP content from 1.3 to 2.0%, and also in AA of the sprouts treated with methyl jasmonate. Świeca [31], after the application of amino acids and elicitor, obtained three-day buckwheat sprouts with a range of TP content from 29.39 to 42.06 mg GAE/g DM. These values were generally lower than those observed in our study, which can be considered high, also in comparison to the polyphenols content of the 26 medicinal and industrial plants in the study by Sytar et al. [36]. The highest content of polyphenols was found in *Stachys byzantina* leaves (18.64 mg GAE/g DM), while Dziadek et al. [37] found 14.08 to 19.53 mg chlorogenic acid equivalent/g DM in the leaves of buckwheat strains.

The TP content in the sprouts of the studied cultivars was affected differently by the PGPs and/or BCAs presence in the treating solutions (Figure 1E). The individual presence of *Ecklonia maxima* extracts and nitrophenols mixture in the treating solution strongly decreased the TP content in Panda sprouts, compared to the untreated sprouts. A similar effect was observed for the sprouts pretreated with nitrophenols mixture and one of the BCAs. On the contrary, a decrease in the TP content was observed for Kora sprouts pretreated with *Bacillus subtilis* alone or in combination with *Ecklonia maxima* extract, when compared to the control.

The content of rutoside in sprouts of both cultivars grown from untreated seeds was comparable (Panada 316 and Kora 318 mg/100 g DM). The application of solutions with different compositions of PGPs and/or BCAs strongly differentiated the content of rutoside in the sprouts of the cultivars studied (Figure 1F). The presence of nitrophenols mixture in the treating solution, either individually or with BCAs, resulted in a decrease of rutoside content in Panda sprouts, and similar effect was noted for Kora sprouts pretreated with *Bacillus subtilis* alone. Suzuki et al. [38] found a significantly higher content of rutoside in the cotyledon of the Tartary buckwheat (50–75 mg/g DM), when compared to our results. Ren and Sun [39] determined 7.60 and 42.24 mg/g of rutoside in nine-day buckwheat and Tartary buckwheat sprouts, respectively. Rutoside content in nine-day buckwheat sprouts was also described by Nam et al. [40] and the level was similar (2.66–3.25 mg/g) to our study. Suzuki et al. [38] declared no presence of rutoside in roots of buckwheat sprouts and only trace amounts in roots of Tartary buckwheat sprouts. In our study, the determination of rutoside content was based on the total weight of the sprout (root, hypocotyl, cotyledon), which may explain the slightly lower concentrations of rutoside.

Differentiation in the content of polyphenolic compounds in buckwheat sprouts pretreated with PGPs and/or BCAs resulted in a relatively low correlation coefficient between TP and rutoside content (0.6341). In the present study, no dependence was particularly observed between TP and rutoside content in Panda sprouts pretreated with *Ecklonia maxima* extract or nitrophenols mixture. This treatment resulted in a significant decrease in TP, but not in rutoside content in the sprouts. On the other hand, the same treatment caused a significant decrease in TP but only a slight decrease in the rutoside content in Kora sprouts (Figure 1E,F). Qin et al. [41] observed an increase in rutoside, isoquercetin, and quercetin content in Tartary buckwheat sprouts treated with sodium bicarbonate, until 96 h, while the content of kaempferol was decreasing up to the 36th hour of sprouts growth.

The content of organic acids in the sprouts of the analyzed buckwheat cultivars varied significantly (Table 2). Panda sprouts contained more oxalic acid, but much less malic acid, as compared to those of Kora cultivar. The presence of oxalic and malic acids in Tartary buckwheat sprouts was confirmed by Peng et al. [42], but without specifying their levels. Statistically significant changes in malic and fumaric acids levels in the sprouts pretreated with PGPs and/or BCAs were noted, while for the other analyzed acids (oxalic, citric, succinic) the observed changes can only be described in terms of a statistical trend.

Analysis of the effect of PGPs and/or BCAs on the content of oxalic acid in the buckwheat sprouts showed a clear growing trend in case when one the treating component was *Bacillus subtilis* (Table 2). This content ranged from 140 to 149.4 mg/100 g DM. On the other hand, the presence of *Ecklonia maxima* extract in the solution favored the accumulation of malic acid, except for the combination with *Pythium oligandrum* oospores. The highest content of this acid was found in the sprouts pretreated with *Ecklonia maxima* extract and nitrophenols mixture (835.2 mg/100 g DM) and was significantly higher than in the untreated sprouts (561.0 mg/100 g DM). The only similar experiment was published by Weber et al. [43], who demonstrated that SiO_2_ and *Ascophyllum nodosum* extract can influence the organic acids content and the sugar/acid ratio in strawberry fruits. The treated strawberries contained significantly fewer organic acids compared to control fruits on the first and third harvest date, and strawberries of both treatments (control and analyzed) were characterized by a higher ratio at later sampling. The correlation between the malic acid content and the AA properties of the sprouts should also be mentioned (Table 3). Apart from the negative correlation between malic acid content and the AA in sprouts determined with the use of DPPH^+^ radical or TPTZ, a negative correlation with TP and a positive correlation with FRAP ratio were confirmed.

Citric acid content in the sprouts was not affected by the treating solutions containing PGPs and/or BCAs, but the content range (217.0–446.3 mg/100 g DM) was large and the majority of the tested treatment combinations showed a tendency to increase its content in the pretreated, in comparison to the untreated sprouts. Moreover, a correlation was shown only between the content of the two of the presented acids, namely citric acid and oxalic acid (r = 0.5888).

The content of succinic acid in the sprouts was also not statistically significant, although it ranged from 368.2 to 629.6 mg/100 g DM. There was a positive correlation between this acid content and AA of buckwheat sprouts (Table 3), and the values of correlation coefficients ranged from 0.1381 to 0.4981, depending on the method of AA determination. We also found a positive correlation between the levels of this acid and TP content (r = 0.4471) and a negative correlation with FRAP ratio (−0.4667).

The content of fumaric acid varied from 66.53 to 87.79 mg/100 g DM, and the differences between the sprouts pretreated with different PGPs and/or BCAs combinations were statistically significant. Treatment with PGPs and/or BCAs in each case resulted in a reduction in the fumaric acid content in the sprouts, as compared to the control. Furthermore, fumaric acid was the only one to show a positive correlation with rutoside (Table 3). 

**Figure 2:**
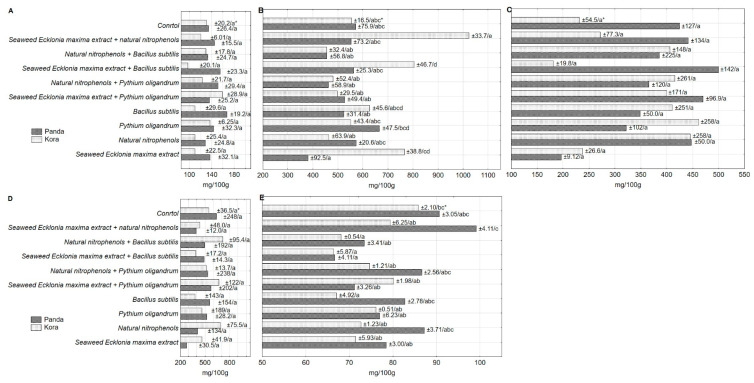
Effect of the treatment on organic acids (oxalic (**A**), malic (**B**), citric (**C**), succinic (**D**), fumaric (**E**)) content in the buckwheat sprouts cvs. Panda and Kora. * standard error of mean/homogeneous groups.

The levels of organic acids, especially malic and fumaric acids, differed between the examined buckwheat cultivars (Figure 2). Control sprouts showed no statistically significant differentiation in the levels of malic acid. Soaking the seeds in the *Ecklonia maxima* extract alone and with the addition of nitrophenols mixture or *Bacillus subtilis* caused a clear increase in the content of malic acid, but only in Kora sprouts (Figure 2B). In case of fumaric acid, the aforementioned combination (*Ecklonia maxima* extract + nitrophenols mixture) increased its content, but only in Panda cultivar. This means that genetically diverse cultivars modify their metabolism differently under the influence of PGPs and/or BCAs, resulting in the different chemical composition of the germ.

In the study of Pereira et al. [44] the effect of biostimulants application on the nutritional quality and bioactive properties of spinach cultivated in protected environment under water stress conditions was evaluated and a variable effect of the tested biostimulants was observed on organic acids of both spinach genotypes.

**Figure 3:**
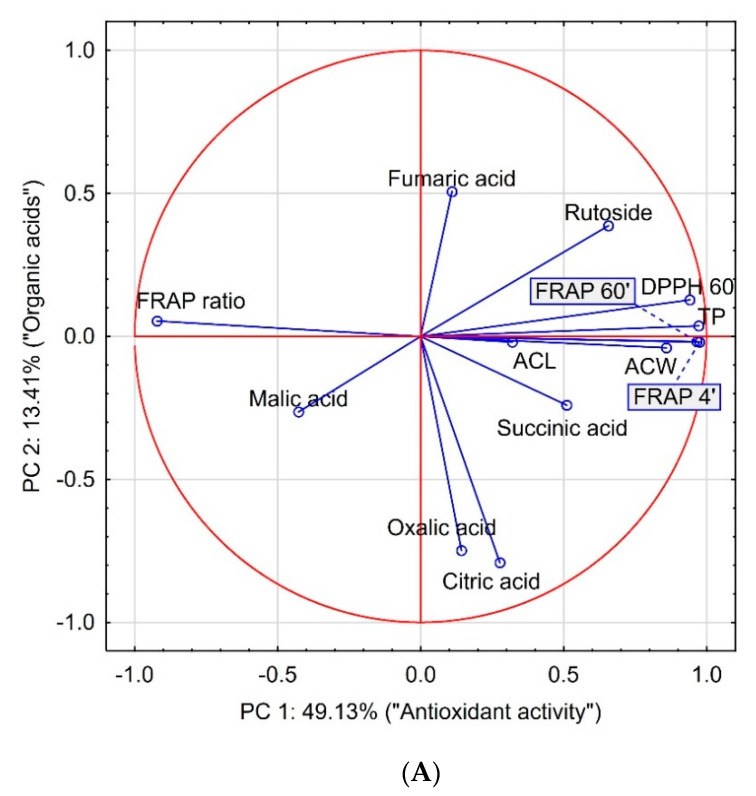

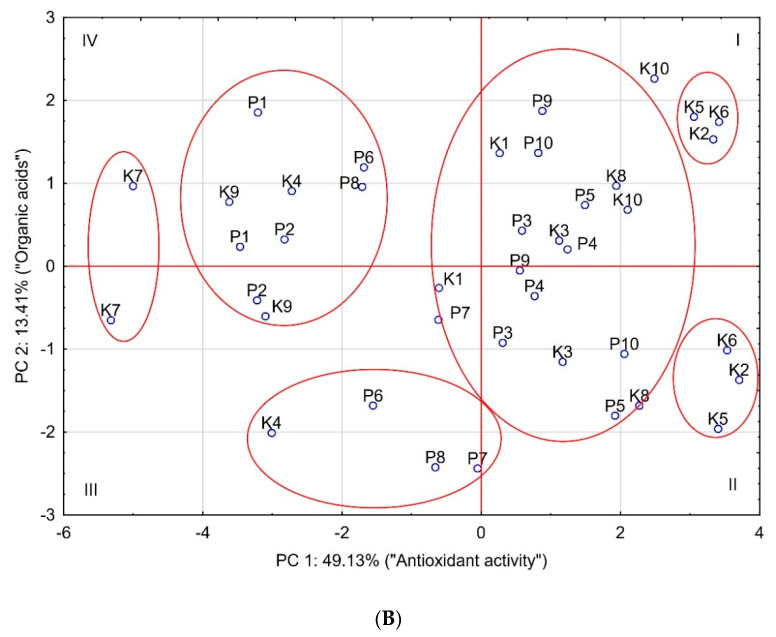
Biplot based on first two principal component axes for chemical composition and antioxidant activity of buckwheat sprouts (**A**) and distribution of two cultivars of buckwheat sprouts based on the first two components obtained from principal component analysis (**B**). K denotes cultivar Kora, P denotes cultivar Panda and numbers from 1 to 10 denote buckwheat seed treatments: 1. Kelpak SL only, 2. Asahi SL only, 3. Polyversum WP only, 4. Serenade ASO only, 5. Kelpak SL plus Polyversum WP, 6. Asahi SL plus Polyversum WP, 7. Kelpak SL plus Serenade ASO, 8. Asahi SL plus Serenade ASO, 9. Asahi SL plus Kelpak SL an 10. Control (untreated, without biostimulants and biological control agents (BCAs)).

Principal component analysis (PCA) indicated that the analyzed parameters concerning chemical content and AA could distinguish the treatment of the buckwheat seeds. Analysis of thirteen chemical parameters was presented on a graph formed by the first two components defined as “antioxidant properties” and “organic acids” (Figure 3). Both components explained 62.54% of the total variation of the original data. The first component was mainly related to the parameters characterizing the antioxidant properties of buckwheat sprouts together with the content of rutoside, while the second component was mainly related to the content of organic acids. The scatterplot diagram on the graph spanning the first two components allowed to group the methods of treating the buckwheat seeds. The first and second quadrants include the treatment of buckwheat seed cultivars that favor the increase in “antioxidant activity”, while the third and fourth quadrants include the treatment of buckwheat seed cultivars generating a high FRAP ratio, negatively correlating with “antioxidant activity” (Figure 3A). On the scatter plot of the case set for the analyzed main components (Figure 3B) the sprouts of Kora cultivar treated with 1. only natural nitrophenols, 2. *Ecklonia maxima* extract + *Pythium oligandrum*, and 3. natural nitrophenols + *Pythium oligandrum* are located on the far right. The variation in the composition of organic acids and rutoside can be observed between the repetitions. Samples localized in the first quarter were richer in rutoside and fumaric acid, while in the second quarter organic acids were predominant components of the sprouts. However, it should be underlined that the contribution of the second principal component explains only 13.41% of the total variance. On the left-hand side of the graph of factor coordinates of cases, the sprouts of Kora cultivar treated with *Ecklonia maxima* extract + *Bacillus subtilis* are observed, with a high FRAP ratio but lower AA.

## 4. Conclusions

Buckwheat sprouts are a valuable source of polyphenols and show high antioxidant activity (AA). We have proved that pretreatment of buckwheat seeds of Kora and Panda cultivars with different combinations of PGPs and/or BCAs can affect their chemical composition and AA. Using various methods of AA determination, a higher antioxidant potential of Kora buckwheat sprouts was confirmed, essentially due to the predominant hydrophilic fraction of antioxidants. The highest AA was obtained in the sprouts grown from seeds soaked in *Ecklonia maxima* extract and *Pythium oligandrum* oospores solution. A higher AA was accompanied by a higher content of TP, but not necessarily a higher content of rutoside. This resulted in high correlation coefficients between AA and TP content and a significantly lower correlation between AA and the rutoside content in buckwheat sprouts. Additionally, principal component analysis (PCA) showed a possibility of indicating a specific method of treating the seeds of individual buckwheat cultivars in order to modify the AA in sprouts. This study complements the gap in knowledge on the AA and nutritional and nutraceutical potential of buckwheat sprouts under the influence of PGPs and/or BCAs.

**Figure 4:**
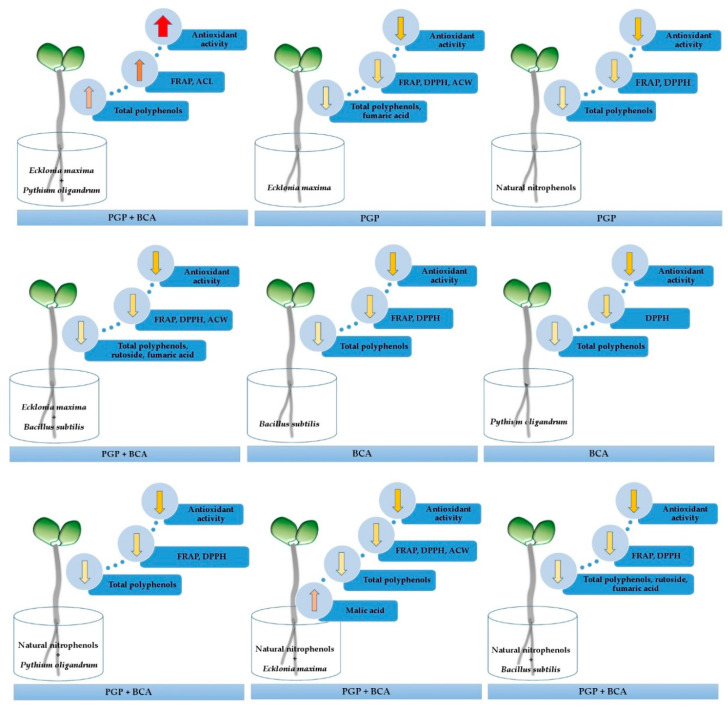
A possible mode of action of plant growth promoters (PGPs) and biological control agents (BCAs) influence on the chemical composition and antioxidant activity of buckwheat sprouts. FRAP—Ferric Reducing Antioxidant Power; DPPH (1,1-diphenyl-2-picrylhydrazyl)—free radical scavenging method for evaluation the antioxidant activity of compounds able to transfer hydrogen atoms; ACL—Lipid-Soluble Antioxidant Capacity; ACW—Water-Soluble Antioxidant Capacity. Up/down arrows indicate an increase/decrease of measured parameters comparing with untreated seeds (control).

In conclusion *Ecklonia maxima* extract together with *Pythium oligandrum* oospores showed synergistic enhancement in antioxidant activity of buckwheat sprouts. Other treatments with PGPs or/and BCAs did not significantly increase antioxidant activity of the sprouts compared to the untreated seeds (control). Based on the results obtained in this study, a possible mode of action of PGPs and BCAs regulation of antioxidant activity due to their influence on polyphenols metabolism is proposed in Figure 4. 

## Figures and Tables

**Table 1:** Effect of cultivar and treatment on antioxidant activity of buckwheat sprouts.

Factor *	Antioxidant Activity					
	FRAP 4′ (µM Fe^2+^/g)	FRAP 60′ (µM Fe^2+^/g)	FRAP Ratio (FRAP 60′/FRAP 4′)	DPPH (μM TRX/g)	ACW (μM AAE/g)	ACL (μM TRX/g)
Cultivar **						
Panda	50.44 ± 1.38 ^a^	99.50 ± 2.29 ^a^	1.973 ± 0.01 ^a^	46.88 ± 1.79 ^a^	163.5 ± 5.82 ^a^	1.132 ± 0.03 ^a^
Kora	54.52 ± 2.99 ^b^	110.00 ± 5.08 ^b^	2.018 ± 0.02 ^b^	52.94 ± 3.68 ^b^	182.7 ± 5.14 ^b^	1.250 ± 0.07 ^b^
Treatment ***						
Seaweed *Ecklonia maxima* extract	39.83 ± 3.10 ^a^	83.03 ± 5.94 ^a^	2.085 ± 0.02 ^c^	41.23 ± 4.34 ^ab^	151.7 ± 19.9 ^a^	1.288 ± 0.07 ^a^
Natural nitrophenols	48.82 ± 5.83 ^c^	98.22 ± 10.4 ^c^	2.012 ± 0.03 ^bc^	52.03 ± 9.94 ^c^	166.6 ± 24.8 ^ab^	1.235 ± 0.15 ^a^
*Pythium oligandrum*	56.18 ± 0.50 ^e^	109.84 ± 1.58 ^de^	1.955 ± 0.02 ^ab^	51.90 ± 2.25 ^c^	184.6 ± 6.05 ^bc^	1.155 ± 0.06 ^a^
*Bacillus subtilis*	53.36 ± 4.39 ^d^	105.38 ± 6.99 ^d^	1.975 ± 0.05 ^ab^	44.10 ± 10.2 ^b^	166.8 ± 2.02 ^ab^	0.993 ± 0.06 ^a^
Seaweed *Ecklonia maxima* extract + *Pythium oligandrum*	62.47 ± 0.72 ^f^	121.31 ± 2.38 ^f^	1.942 ± 0.02 ^a^	56.33 ± 3.91 ^c^	195.6 ± 6.82 ^c^	1.568 ± 0.24 ^b^
Natural nitrophenols + *Pythium oligandrum*	52.84 ± 5.69 ^d^	105.22 ± 10.5 ^d^	1.991 ± 0.02 ^ab^	54.68 ± 7.54 ^c^	174.8 ± 16.3 ^b^	1.115 ± 0.06 ^a^
Seaweed *Ecklonia maxima* extract + *Bacillus subtilis*	43.92 ± 4.06 ^b^	90.25 ± 6.29 ^b^	2.055 ± 0.06 ^c^	37.28 ± 7.26 ^a^	153.8 ± 9.59 ^a^	1.215 ± 0.06 ^a^
Natural nitrophenols + *Bacillus subtilis*	53.03 ± 3.62 ^d^	104.73 ± 7.19 ^d^	1.975 ± 0.02 ^ab^	52.68 ± 3.75 ^c^	175.8 ± 7.50 ^b^	1.063 ± 0.03 ^a^
Seaweed *Ecklonia maxima* extract + natural nitrophenols	49.07 ± 3.30 ^c^	99.10 ± 4.47 ^c^	2.020 ± 0.05 ^bc^	43.68 ± 6.63 ^b^	175.1 ± 10.1 ^b^	1.062 ± 0.03 ^a^
Control	58.48 ± 1.40 ^e^	112.91 ± 2.64 ^e^	1.931 ± 0.02 ^a^	64.98 ± 5.18 ^d^	186.1 ± 5.22 ^bc^	1.218 ± 0.07 ^a^

* means were compared separately within the factors (1. cultivar and 2. treatment); ** the cultivar means denoted by different letters differ statistically at α = 0.05 (for all columns separately); *** the treatment means denoted by different letters differ statistically at α = 0.05 (for all columns separately).

**Table 2:** Effect of cultivar and treatment on total polyphenols, rutoside, and organic acids content in buckwheat sprouts.

Factor *	Ingredient						
	Total Polyphenols (mg GAE/g)	Rutoside (mg/100 g)	Oxalic Acid (mg/100 g)	Malic Acid (mg/100 g)	Citric Acid (mg/100 g)	Succinic Acid (mg/100 g)	Fumaric Acid (mg/100 g)
Cultivar **							
Panda	40.42 ± 1.56 ^a^	280.1 ± 9.51 ^a^	144.0 ± 6.51 ^a^	527.2 ± 20.8 ^a^	388.1 ± 32.4 ^a^	490.7 ± 42.9 ^a^	81.38 ± 2.22 ^b^
Kora	43.42 ± 2.04 ^b^	305.6 ± 8.12 ^b^	124.5 ± 6.35 ^a^	622.7 ± 40.3 ^b^	343.1 ± 44.8 ^a^	537.4 ± 34.1 ^a^	74.87 ± 1.61 ^a^
Treatment ***							
Seaweed *Ecklonia maxima* extract	35.52 ± 4.71 ^b^	311.2 ± 16.8 ^cd^	123.4 ± 17.8 ^a^	572.9 ± 118 ^abc^	217.0 ± 16.6 ^a^	368.2 ± 58.2 ^a^	74.94 ± 3.38 ^ab^
Natural nitrophenols	41.67 ± 5.63 ^d^	283.3 ± 17.3 ^abc^	119.2 ± 15.4 ^a^	517.6 ± 42.0 ^ab^	446.3 ± 107 ^a^	548.5 ± 102 ^a^	79.92 ± 4.50 ^bc^
*Pythium oligandrum*	44.62 ± 0.58 ^e^	306.4 ± 13.7 ^cd^	140.0 ± 13.6 ^a^	608.9 ± 42.4 ^bc^	391.8 ± 120 ^a^	492.2 ± 79.7 ^a^	76.57 ± 2.56 ^abc^
*Bacillus subtilis*	40.16 ± 4.26 ^c^	265.6 ± 15.9 ^abc^	144.0 ± 19.8 ^a^	564.8 ± 33.9 ^abc^	373.4 ± 85.3 ^a^	482.0 ± 106 ^a^	76.48 ± 4.43 ^abc^
Seaweed *Ecklonia maxima* extract + *Pythium oligandrum*	49.03 ± 0.71 ^g^	342.4 ± 7.52 ^d^	149.4 ± 18.6 ^a^	508.9 ± 23.6 ^ab^	428.0 ± 100 ^a^	629.4 ± 95.6 ^a^	76.55 ± 2.66 ^abc^
Natural nitrophenols + *Pythium oligandrum*	44.28 ± 5.49 ^e^	299.4 ± 25.7 ^abc^	136.9 ± 17.0 ^a^	471.3 ± 32.6 ^ab^	390.6 ± 118 ^a^	525.2 ± 97.6 ^a^	80.65 ± 3.65 ^bc^
Seaweed *Ecklonia maxima* extract + *Bacillus subtilis*	34.16 ± 4.07 ^a^	253.0 ± 8.81 ^ab^	125.9 ± 20.8 ^a^	683.8 ± 73.2 ^c^	340.8 ± 109 ^a^	438.1 ± 29.7 ^a^	66.53 ± 2.93 ^a^
Natural nitrophenols + *Bacillus subtilis*	44.14 ± 3.55 ^e^	241.1 ± 30.7 ^a^	130.9 ± 12.5 ^a^	449.7 ± 25.4 ^a^	397.9 ± 108 ^a^	629.6 ± 96.7 ^a^	70.23 ± 1.73 ^ab^
Seaweed *Ecklonia maxima* extract + natural nitrophenols	38.99 ± 3.88 ^c^	309.3 ± 17.0 ^cd^	130.4 ± 8.44 ^a^	835.2 ± 119 ^d^	339.4 ± 73.0 ^a^	419.4 ± 28.8 ^a^	87.27 ± 6.07 ^c^
Control	46.62 ± 0.56 ^f^	316.9 ± 16.1 ^cd^	131.6 ± 13.9 ^a^	561.0 ± 26.0 ^abc^	308.8 ± 69.0 ^a^	582.4 ± 83.8 ^a^	87.79 ± 1.91 ^c^

* means were compared separately within the factors (1. cultivar and 2. treatment); ** the cultivar means denoted by different letters differ statistically at α = 0.05 (for all columns separately); *** the treatment means denoted by different letters differ statistically at α = 0.05 (for all columns separately).

**Table 3:** Correlation coefficients and their levels of significance (*p*).

Item	FRAP 4′	FRAP 60′	TP	DPPH	ACW	ACL	FRAP Ratio	Rutoside	Oxalic Acid	Malic Acid	Citric Acid	Succinic Acid
FRAP 60′	0.9978 *p* = 0.000	1.0000 *p* = ---										
TP	0.9645 *p* = 0.000	0.9609 *p* = 0.000	1.0000 *p* = ---									
DPPH	0.8990 *p* = 0.000	0.8853 *p* = 0.000	0.9063 *p* = 0.000	1.0000 *p* = ---								
ACW	0.8182 *p* = 0.000	0.8207 *p* = 0.000	0.8837 *p* = 0.000	0.8100 *p* = 0.000	1.0000 *p* = ---							
ACL	0.2682 *p* = 0.094	0.2730 *p* = 0.088	0.2000 *p* = 0.216	0.2900 *p* = 0.069	0.2550 *p* = 0.112	1.0000 *p* = ---						
FRAP Ratio	−0.8897 *p* = 0.000	−0.8609 *p* = 0.000	−0.8847 *p* = 0.000	−0.8715 *p* = 0.000	−0.7190 *p* = 0.000	−0.1697 *p* = 0.295	1.0000 *p* = ---					
Rutoside	0.5990 *p* = 0.000	0.6033 *p* = 0.000	0.6341 *p* = 0.000	0.6280 *p* = 0.000	0.5409 *p* = 0.000	0.3053 *p* = 0.055	−0.4832 *p* = 0.002	1.0000 *p* = ---				
Oxalic acid	0.1230 *p* = 0.449	0.1091 *p* = 0.503	0.0798 *p* = 0.625	0.0580 *p* = 0.722	0.0127 *p* = 0.938	0.1425 *p* = 0.380	−0.2425 *p* = 0.132	−0.0552 *p* = 0.735	1.0000 *p* = ---			
Malic acid	−0.3870 *p* = 0.014	−0.3593 *p* = 0.023	−0.3551 *p* = 0.025	−0.4268 *p* = 0.006	−0.1180 *p* = 0.468	−0.1603 *p* = 0.323	0.5141 *p* = 0.001	−0.1751 *p* = 0.280	0.0488 *p* = 0.765	1.0000 *p* = ---		
Citric acid	0.2353 *p* = 0.144	0.2243 *p* = 0.164	0.2286 *p* = 0.156	0.1486 *p* = 0.360	0.2797 *p* = 0.080	0.0165 *p* = 0.920	−0.3043 *p* = 0.056	−0.0617 *p* = 0.705	0.5888 *p* = 0.000	−0.0324 *p* = 0.842	1.0000 *p* = ---	
Succinic acid	0.4981 *p* = 0.001	0.4889 *p* = 0.001	0.4471 *p* = 0.004	0.3939 *p* = 0.012	0.3618 *p* = 0.022	0.1381 *p* = 0.396	−0.4667 *p* = 0.002	0.1369 *p* = 0.399	0.0604 *p* = 0.711	−0.1160 *p* = 0.476	0.2256 *p* = 0.162	1.0000 *p* = ---
Fumaric acid	0.0084 *p* = 0.959	−0.0149 *p* = 0.927	0.0896 *p* = 0.583	0.1622 *p* = 0.317	−0.0231 *p* = 0.887	−0.0727 *p* = 0.656	−0.1887 *p* = 0.244	0.3119 *p* = 0.050	−0.0602 *p* = 0.712	−0.2532 *p* = 0.115	−0.1352 *p* = 0.406	−0.0848 *p* = 0.603
